# Update on Hepatitis E Virus Infection 2025: Insights From an International Symposium

**DOI:** 10.1111/liv.70364

**Published:** 2025-10-17

**Authors:** Lara Buchardt, Viet Loan Dao Thi, Reimar Johne, Mathias Ziersch, Dominik Harms, Jörg Hofmann, Katja Dinkelborg, Mara Klöhn, Mira Choi, Jens Kurreck, Heiner Wedemeyer, Daniel Todt

**Affiliations:** ^1^ Department of Translational and Computational Infection Research Ruhr University Bochum Bochum Germany; ^2^ Department of Molecular and Medical Virology Ruhr University Bochum Bochum Germany; ^3^ Schaller Research Group, Department of Infectious Diseases, Virology Heidelberg University, Medical Faculty Heidelberg Heidelberg Germany; ^4^ Department of Biological Safety German Federal Institute of Risk Assessment Berlin Germany; ^5^ Department of Applied Biochemistry, Institute for Biotechnology Technische Universität Berlin Berlin Germany; ^6^ Department of Infectious Diseases Robert Koch Institute Berlin Germany; ^7^ Labor Berlin, Charité‐Vivantes GmbH Berlin Germany; ^8^ TWINCORE, Centre for Experimental and Clinical Infection Research, A Joint Venture Between Helmholtz‐Centre for Infection Research and Hannover Medical School Hannover Germany; ^9^ Department of Gastroenterology, Hepatology, Infectious Diseases and Endocrinology Hannover Medical School Hannover Germany; ^10^ German Center for Infectious Disease Research (DZIF); Partner Site Hannover‐Braunschweig Hannover Germany; ^11^ Hepatitis E Virus Research Hub (HepE‐Hub) Bochum Germany; ^12^ Department of Nephrology and Medical Intensive Care Charité Universitätsmedizin Berlin Berlin Germany; ^13^ Cluster of Excellence RESIST (EXC2155) Hannover Medical School Hannover Germany; ^14^ Leberstiftungs‐GmbH Deutschland Hannover Germany; ^15^ European Virus Bioinformatics Center (EVBC) Jena Germany

**Keywords:** antivirals, hepatitis E virus, neutralising antibodies, ratHEV

## Abstract

**Background and Aims:**

The second Hepatitis E Symposium was held as an integral part of the International DFG/DZIF Joint Meeting on Viral Infections of the Liver and the Heart in Berlin. The symposium aimed to provide a comprehensive exploration of hepatitis E virus (HEV) research and bring together clinicians, basic scientists, public health experts, and bioinformaticians from around the world.

**Methods:**

The program comprised two focused thematic sessions with eight invited speakers presenting recent advances in HEV biology, pathogenesis, and clinical impact. In addition, two interactive round table discussions and two poster sessions facilitated the exchange of recent findings and fostered critical debate.

**Results:**

Core topics included current challenges in HEV epidemiology, emerging therapeutic strategies, and advances in the clinical management of HEV infections. The round table discussions fostered intensive interdisciplinary dialogue, identifying key knowledge gaps as potential avenues for collaborative research.

**Conclusion:**

This report summarises the data presented at the symposium and outlines the main themes and outcomes of the round table discussions, highlighting both the progress made and the future directions for HEV research.

**Summary**

- This meeting brought together experts from around the world to share the latest knowledge on hepatitis E, a virus that can cause liver disease.
- Researchers discussed viral spread and species barriers, how to improve diagnosis and treatment, and where important knowledge gaps remain.
- The event encouraged collaboration to develop better ways to prevent and manage hepatitis E infections in the future.

## 1 Introduction

Despite the significant public health burden imposed by the hepatitis E virus (HEV), with almost 19.5 million infections and 3500 deaths in 2021 globally, the virus remains insufficiently studied [1, 2]. Eight different HEV genotypes have been identified, of which the genotypes 1–4 (HEV1‐ HEV4) are most common to cause human infections The mode of transmission differs between these genotypes. HEV1 and HEV2 are mostly present in resource‐limited countries, transmitted via the faecal‐oral route, and outbreaks often occur through contaminated water [1]. In high‐income countries, HEV3 and HEV4 are most common, which are transmitted zoonotically through the consumption of undercooked meat of infected animals like pigs, deer, or rabbits [1, 3]. In addition, rat HEV, which is only distantly related to HEV1–4 and mainly distributed in rats, has recently been identified as a zoonotic pathogen capable of causing hepatitis in humans [4].

Approximately 70%–95% of all HEV infections are asymptomatic or present with only mild symptoms [5]. Nevertheless, an HEV infection can cause acute hepatitis characterised by symptoms like nausea, vomiting, abdominal pain, jaundice, fever or malaise [6]. While healthy individuals mostly clear HEV infection without further complications, immunocompromised patients, like solid organ transplant recipients, can develop a chronic HEV infection. Furthermore, pregnant women have an increased risk of developing a fulminant HEV infection, which can lead to maternal mortality or fetal abnormalities in many cases [2, 7]. Currently, there is no approved treatment for an HEV infection. In cases where the HEV infection is cleared spontaneously, no treatment is required. In cases of a fulminant hepatitis, the off‐label use of the nucleoside analogue ribavirin is recommended. Unfortunately, this antiviral medication is contraindicated in pregnant women, as it is teratogenic and can cause severe anaemia in the context of renal impairment. Given that chronic HEV infections predominantly affect immunocompromised patients, the primary treatment strategy entails the reduction of immunosuppressive therapy, if feasible. Additionally, the administration of PEGylated‐interferon‐α can be considered for liver transplant recipients, if the therapy with ribavirin does not lead to the clearance of the virus [8, 9].

Overall, knowledge gaps regarding the clinical management of HEV and molecular mechanisms of the virus still exist, highlighting the importance of connecting international researchers and clinicians working on HEV to exchange current research findings and clinical strategies for an HEV infection.

The second Hepatitis E Symposium was held in Berlin and brought together international researchers to discuss the latest advances in HEV research. In addition to participants from Europe, the United States, and Australia, a notably high number of researchers and clinicians from Vietnam and Nigeria—where HEV infections pose a significant public health burden—were able to attend. The symposium covered a wide range of topics, including basic virology, the development of new treatment strategies, clinical treatment recommendations, and epidemiology.

## 2 Outcome Prediction and Personalised Medicine for Hepatitis E

### 2.1 Potential Reservoir of HEV Infection in the Intestinal Stem Cell Niche (By Dr. Viet Loan Dao Thi)

**FIGURE 1:**
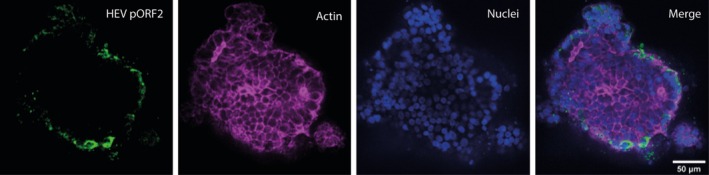
Human pluripotent stem cell‐derived intestinal organoids (hIOs) were infected with cell culture derived HEV3 (7 × 10^6^ GE/well) and stained for ORF2 HEV protein (green), Actin (magenta), and nuclei (blue) at day 14 post‐infection. Sample was imaged with a spinning Disc Ti2 CREST (Nikon) and a 20X air objective. Image was processed using ImageJ software.

As HEV is transmitted enterically and is often ingested via contaminated food or water, it has to overcome the intestinal barrier in order to reach the liver, which is the main replication site of the virus [1, 8]. Up to now, it is not clear if HEV can infect the intestinal barrier or if it simply passes it via transcytosis or other modes of translocation. Two studies already suggested that HEV can replicate in intestinal cells [10, 11], which motivated the group of Dr. Dao Thi to investigate this potential replication site further. To do so, the group established intestinal organoids (hIOs), derived from induced pluripotent stem cells (Figure 1). These organoids represent the cell heterogenicity of the human intestine and have a stem cell crypt containing proliferating cells. The group was able to infect hIOs with cell culture‐ and patient‐derived HEV and showed that the infection is primarily maintained in the crypts of organoids (Figure 1). This viral replication in the intestinal crypts was also found in intestinal biopsies from HEV patients. Compared to HAV, which was cleared from the hIOs within 4 days, HEV was able to persist in hIOs for up to 40 days, likely through cell division‐mediated spread. Dr. Dao Thi concluded that the intestine should be regarded as a potential reservoir for HEV infection and this should be considered in the development of future therapeutics.

### 2.2 Zoonotic Potential of Rat Hepatitis E Virus in Germany (By Prof. Dr. Reimar Johne)

While HEV1 and HEV2 only infect humans and are transmitted via the faecal‐oral route, for example due to the consumption of contaminated water, other HEV strains can infect humans via zoonotic infections. It is broadly known that HEV3 and HEV4 infect domestic pigs and wild boars and can be transmitted to humans via the consumption of contaminated meat products [12]. In the early 2000s it has been suspected that rats can also be infected with HEV. In 2010, Reimar Johne and colleagues could show that distinct HEV strains are circulating in rats in Hamburg, which are only distantly related to the HEV‐1–4 genotypes and rather cluster in a separate group [13, 14]. During the following years, this virus was found in rats all over the world, and the phylogenetic analysis led to the classification of rat HEV as a separate species *Rocahepevirus ratti* (HEV‐C) [15, 16, 17]. Since the identification of the virus, it has been identified globally in rats, with most reports from Asian and European countries. Two distinct genotypes of the virus have been described, termed HEV‐C1 and ‐C2, which occur in different host species [15, 18]. Since 2018, several human rat HEV infections, most of which occurred in Hongkong and Spain, and often connected with symptomatic hepatitis, have been described [19, 20, 21]. This motivated Prof. Johne to continue his research on rat HEV in Germany. The human cell line Huh7‐Lunet‐BLR is susceptible to replication of rat HEV with no obvious differences in the replication of strains isolated from rat and human of Germany and Hongkong, indicating a general zoonotic potential of rat HEV strains [22]. After sampling wild rats in Berlin in 2023, rat HEV could be identified in 8.4% of the specimens. This number was similar to the 11.5% of rats in Berlin, which were tested positive for rat HEV in 2009/2010, indicating continued circulation of the virus in this wild rat population. In an analysis of 1000 human samples from the Charité—Universitätsmedizin Berlin, one of the analysed samples was positive for rat HEV RNA, which proved that human infections of rat HEV also occur in Germany although with a low detection rate (0.1%) [23]. Taken together, rat HEV continuously circulates in wild rats, which represent a permanent source of infection for humans, even though infections obviously occur at a low incidence in Germany.

### 2.3 RNA Interference Strategies Targeting HEV—Combining RNA Interference and RIG‐I Activation to Inhibit Hepatitis E Virus Replication (By Mathias Ziersch)

**FIGURE 2:**
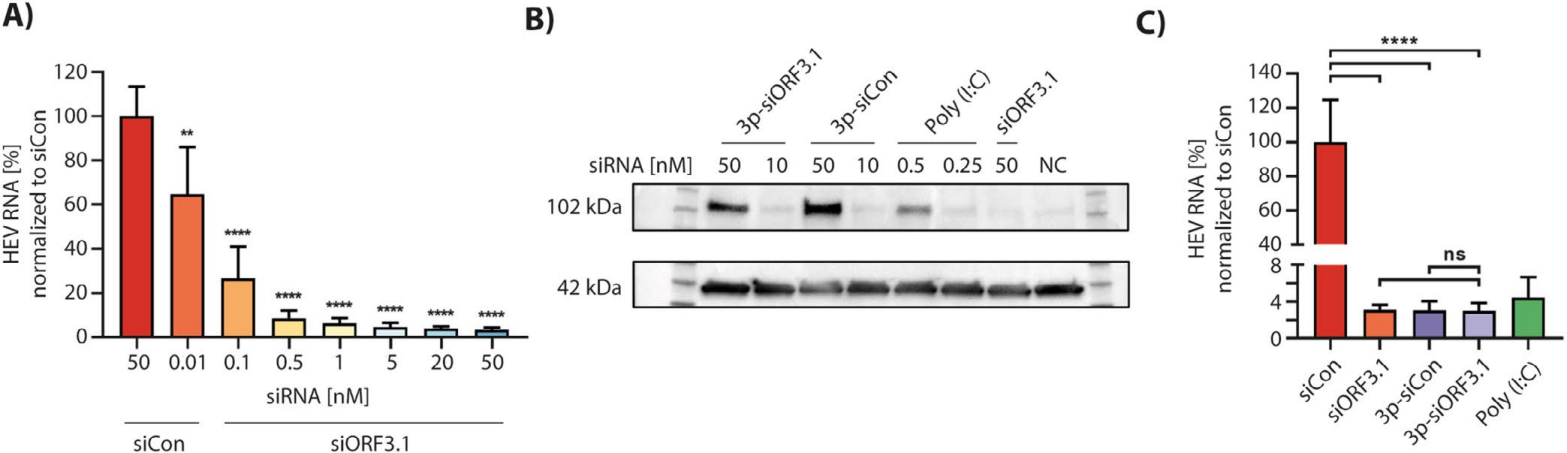
Utilising a dual approach of RNA interference and RIG‐I activation to suppress hepatitis E virus replication in persistently infected A549/pers‐HEV cells and in A549/D3 cells prior to infection. (A) Inhibition of HEV replication by siRNA in infected A549/D3 cells. Cells were transfected with varying concentrations (0.01–50 nM) of siORF3.1, and 24 h later, infected with HEV isolates at a multiplicity of infection (MOI) of 1.0 for 16 h. (B) Evaluation of RIG‐I expression by western blot. A549/D3 cells were stimulated for 48 h with different concentrations of 3p‐siCon, 3p‐siORF3.1 (10 and 50 nM), poly I:C (0.25 and 0.5 ng/mL), or siORF3.1 (50 nM). Non‐transfected A549/D3 cells served as a negative control (NC), and Actin was used as a loading control. (C) Inhibition of HEV replication by 3p‐siRNA. A549/D3 cells were transfected with the indicated siRNAs (siCon, siORF3.1, 3p‐siCon, 3p‐siORF3.1) at 50 nM. HEV infection (MOI 1.0) was performed 24 h post‐transfection and continued for 16 h. Mean ± SD of three independent experiments (*n* = 3) are shown. ns = not significant, ***p ≤ 0.001, ****p ≤ 0.0001. The figure is adapted from [22] used under a CC BY 4.0 International Licence.

RNA‐Interference (RNAi) is the process in which short, double‐stranded RNA segments (siRNA) interact with the Ago2 protein to form the RISC complex, which targets RNA complementary to the siRNA and cleaves it [24]. There are several reasons why the utilisation of RNAi can be considered as a treatment option for HEV. Firstly, siRNA can be modified with N‐Acetylgalactosamin, which binds to the asiaglycoprotein receptor. This receptor mediates endocytosis in liver cells, especially under cirrhotic conditions. Therefore, the siRNA is delivered to the liver efficiently [25]. Additionally, phase II studies have already shown successes in treating a Hepatitis B virus infection with siRNA [26, 27]. Mathias Ziersch decided on different siRNAs, targeting either ORF3 of the HEV RNA, as this is a very conserved region, or the viral helicase, as a control, to test for their anti‐HEV capacity. Two of the three siRNAs targeting ORF3 efficiently reduced HEV RNA copy numbers in persistently and acutely infected cell lines. The optimal effect could be observed 96 h after a dual siRNA transfection within 24 h. The reduction of the HEV RNA was dose‐dependent and already occurred at very low doses in the sub‐nanomolar range (Figure 2) [28]. To improve the anti‐viral effect, a 5′‐triphosphate modification was covalently coupled to the siRNA to activate RIG‐I, a cytoplasmic pattern recognition receptor that recognises viral RNA. It was demonstrated that induction of RIG‐I effectively suppresses HEV replication [29]. Although the dual‐action strategy, combining activation of the adaptive immune response with stimulation of the intrinsic RNAi pathway, did not yield additive effects, this approach may help prevent the emergence of escape mutants resistant to siRNA‐mediated silencing (Figure 2). In the future, the group wants to test 3D organ models to evaluate the performance of the siRNA against HEV [30] and they want to combine their siRNA treatment with the administration of ribavirin derivates to test the antiviral potential of combination therapy.

### 2.4 Dynamics of Hepatitis E Virus Variant Evolution During Ribavirin Treatment of Chronic Infection: An In‐Depth In Vitro Versus In Vivo Comparison (By Dr. Dominik Harms)

Chronic HEV infection may manifest in immunocompromised patients, indicated by the persistence of HEV RNA in blood or stool for more than 3 months. As there is currently no specific therapy for HEV, treatment options are limited to off‐label administration of ribavirin [7, 31]. However, past studies have described selection of viral variants under ribavirin administration that are associated with treatment failure [32]. Dr. Harms presented the case of a kidney transplant patient, suffering from chronic HEV infection since 2009. The patient was repeatetly treated with ribavirin, achieving no sustained virological response. During treatment, Sanger sequencing of the HEV polymerase region was performed multiple times, demonstrating the emergence of multiple ribavirin‐associated viral variants. Known and previously unreported variants were identified by deep sequencing of the complete viral genome at time points before and after treatment discontinuation.

To further analyse the evolutionary dynamics of ribavirin‐associated variants, Dr. Harms used a cell line persistently infected with an HEV isolate from the presented patient [33]. After multiple rounds of antiviral treatment, viral response to ribavirin declined and specific viral variants became dominant within the quasispecies. By comparing patient‐ and culture‐specific variants, several common ORF1 and ORF2 mutations were identified. Some of these variants have already been described in the context of ribavirin while others were newly identified in this study.

Dr. Harms concluded that the periodical ribavirin treatment promotes the emergence and selection of treatment failure‐associated viral variants and therefore a high‐dose prolonged treatment might be beneficial. Additionally, one should consider the impact of other viral variants in the context of treatment failure other than those that are already known.

### 2.5 Round Table Discussion: Research in HEV—Key Challenges and Future Perspectives

Following each session of scientific presentations, a round table discussion provided an opportunity for interactive dialogue, critical feedback, and interdisciplinary exchange.

In the course of the discussion, various challenges regarding HEV were debated, which are presented here in summary.

#### 2.5.1 Rat HEV

Much is still unknown about rat HEV. Up to now, the virus has only been detected in a limited number of host species, namely rats, humans, pigs, and a brown bear [20, 21, 34, 35]. Furthermore, chickens have successfully been artificially infected with rat HEV [36]. In order to properly study rat HEV and reliably detect human infections, diagnostic tests for the detection of rat HEV are urgently needed. Currently, molecular assays are largely missing, leading to an incomplete picture of human rat HEV cases [37, 38].

The zoonotic potential of rat HEV was evidenced by the detection of human infections with this virus. As for now, the burden of spillover events and the risk to human health have yet to be ascertained. Therefore, further research on rat HEV and its species barrier is necessary [21, 39]. In order to study the virus, its species barriers and host restriction factors, innovative methods, like the organoid model presented earlier, could potentially be employed [40, 41].

#### 2.5.2 Prevention of HEV Infections

To reduce the burden of HEV, preventive measures are crucial. Hecolin is the only commercially available vaccine against HEV. This vaccine is currently only approved in China and is mainly based on the HEV1. A recent study assessed the effectivity of Hecolin against HEV3 in a pig infection model. In this model, Hecolin did not confer a sterilising immunity against HEV3 [42].

As pigs serve as a host for HEV and are often the source of zoonotic infections, a One Health approach could also be beneficial to prevent human infections with HEV3 and 4. Hence, some studies aimed to identify vaccines to immunise pigs against HEV, potentially reducing the number of zoonotic HEV infections in humans [43].

Currently, the most effective measure to prevent zoonotic HEV infections is the proper heating of pork products before consumption, to thermally inactivate HEV [44].

#### 2.5.3 Challenges in HEV Research That Need to Be Addressed

At the end of the first round table discussion, the panellists named current challenges they want to address with their HEV research. The entry receptor as well as other host cell factors for the HEV replication is still unknown. These factors can be valuable targets for the development of antiviral medications and therefore, further research is required in this field [45].

In high‐income countries, HEV is mainly transmitted through contaminated food. Due to the high number of zoonotic infections through pigs, pork products are a high‐risk food for acquiring HEV. It is important to identify further food‐borne sources of HEV infections in order to prevent zoonotic HEV infections [46] in a One Health approach [47].

Hecolin is the only commercially available vaccine against HEV and only approved in China. The identification of further vaccines against HEV would be crucial to reduce infections. Ideally, this vaccine would be effective against different HEV genotypes to guarantee a broad protection [48].

Finally, it will be important to further study rat HEV and its zoonotic potential in order to evaluate the risk of this disease for humans [21, 39].

## 3 Diagnostics and Novel Therapeutics for Hepatitis E Virus Infections

### 3.1 The Hepatitis E Virus—More Than Just the 5th Wheel on the Wagon (By Prof. Dr. Jörg Hofmann)

As the number of reported HEV cases is continuously increasing in Germany, there is a great need for reliable diagnostic tools for HEV [49]. Especially, the detection of chronic HEV cases presents clinical challenges as the time of the appearance of first antibodies can vary, liver enzymes are only moderately elevated, and IgM can often be detected for a very long time period [7]. In a comparative analysis, tests for the detection of HEV IgM as well as IgG antibodies performed well in terms of specificity and sensitivity [50]. Nevertheless, factors like the timing of sample collection, the patient's immune status, and the HEV genotype can influence test performance. Therefore, the European Association for the Study of the Liver (EASL) recommends the combination of testing for anti‐HEV antibodies as well as HEV nucleic acid to detect HEV infections [7]. Meanwhile, there is no point‐of‐care test approved by the FDA or prequalified by the WHO, and therefore the establishment of these tests is required by each institution separately [51].

The Labor Berlin analysed samples from more than 15 000 patients in order to evaluate clinical testing of HEV for acutely and chronically infected patients. They found that regarding IgG and HEV RNA levels, but not IgM levels, there were significantly more male individuals that tested positive. Furthermore, they found that the HEV viral load is significantly higher in chronically infected individuals than in acutely infected patients. Based on this data, the Labor Berlin comprised recommendations to identify acute and chronic HEV infections in immunocompetent and immunocompromised patients for their sender clientele (Table 1). Lastly, Prof. Hofmann highlighted that it is not sufficient to analyse transaminase levels for viral monitoring, as these liver values are reduced by a Ribavirin treatment, even if the viral loads are still high.

**TABLE 1:** Strategy to identify an HEV infection.

HEV‐IgG	HEV‐IgM	HEV‐RNA	Interpretation	Recommendation for the sender clientele
Immunocompetent patients				
Negative	Negative		No infection	Immunoblot and/or RT‐PCR
Negative	Positive		Unspecific IgM?	
Positive	Positive		Suspected acute infection	
Positive	Negative		Past infection	
Immunocompromised patients				
Positive	Negative	Positive	Florid infection	PCR follow‐up after 12 weeks or more frequently if symptoms occur. No further antibody diagnostics necessary.
Positive	Positive	Positive		
Positive	Positive	Negative	recently resolved; false positive IgM reactivity	
Negative	Positive	Positive	Florid infection	PCR follow‐up after 12 weeks or more frequently if symptoms occur. Optional: Antibody follow‐up until seroconversion.
Negative	Negative	Positive		

*Note:* Diagnostic workflow for identifying an acute, past or chronic infection in immunocompetent and immunocompromised patients.

### 3.2 A Novel Class of Human Monoclonal Antibodies Neutralising HEV (By Dr. Katja Dinkelborg)

**FIGURE 3:**
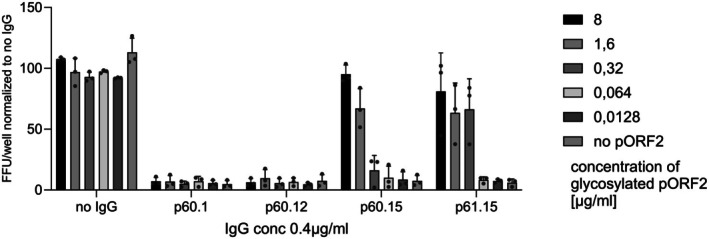
Neutralisation of HEV‐3 and indicated antibodies after pre‐incubation of rising concentrations of glycosylated pORF2 dimers. Focus forming units per well (FFU/well) are displayed normalised to the control. The figure is adapted from [53] used under a CC BY 4.0 International Licence.

HEV infections are the most common cause of acute viral hepatitis and can lead to severe outcomes in patients with risk factors like pregnancy or an underlying liver disease [2, 6, 52]. Nevertheless, the therapeutic options are limited to the off‐label use of Ribavirin and therefore it is important to find direct antivirals to treat HEV infections. In collaboration with Dr. Krey from the University of Lübeck, Dr. Dinkelborg and the research group of Dr. Patrick Behrendt isolated memory B‐cells binding the HEV capsid from convalescent HEV patients. These human antibodies were tested for their HEV binding and neutralisation. In cell culture experiments, the antibody candidates neutralised non‐enveloped HEV particles with different efficiencies, with some also displaying a neutralising effect against the pseudo‐enveloped virus (Figure 3) [53]. An important consideration when designing neutralising antibodies against HEV is that the ORF2 protein, which forms the capsid of the virus, occurs in a glycosylated form. This glycosylated ORF2 protein is likely shed by infected cells to capture anti‐HEV antibodies and therefore avoid virus neutralisation [54, 55]. Of the different antibody candidates, there were at least two glycan‐sensitive antibodies that only bound the infectious ORF2 protein but not the glycosylated form of the protein. These glycan‐sensitive antibodies were able to neutralise HEV even in the presence of high amounts of the glycosylated ORF2 (Figure 3).

As the tested antibodies bind to a conserved domain of the viral protein, the glycan‐sensitive antibodies also readily neutralise HEV1–4 as well as rat HEV. These antibodies also protect animals in different infection settings from an HEV infection.

In the future, these neutralising antibodies could potentially present a therapeutic option for HEV infections and serve as a useful tool in HEV research [53].

### 3.3 Novel Antivirals to Combat HEV Infections (By Dr. Mara Klöhn)

**FIGURE 4:**
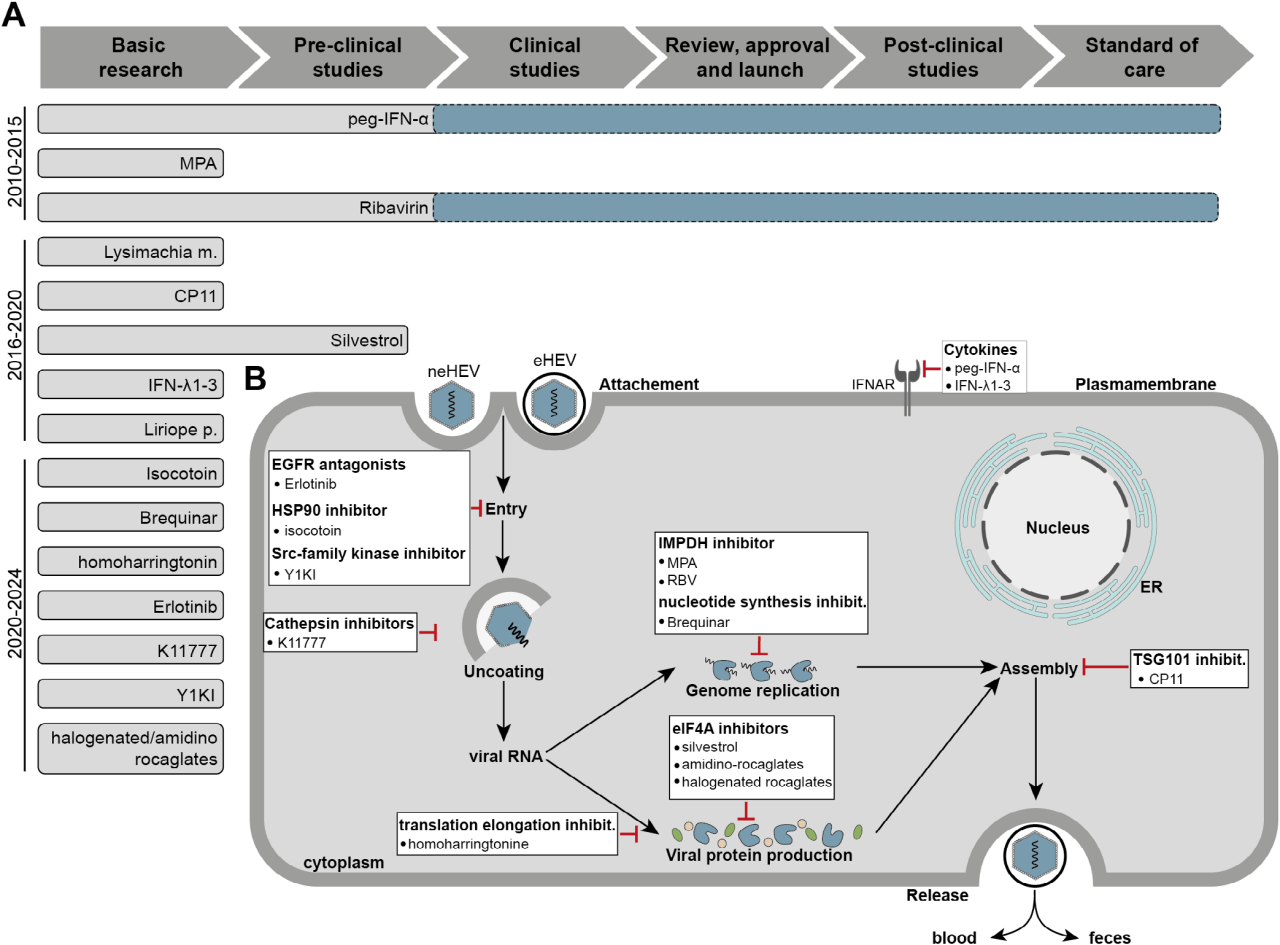
Recent advances in drug development for HEV with a focus on host‐targeting antivirals. (A) Shown are antivirals against HEV and their current developmental stage. The time of identification of the agents is indicated on the left. Ribavirin and pegylated α (peg‐IFN‐α) are applied off‐label for HEV treatment, lacking formal clinical trial validation for this indication (shown as dashed lines). (B) Illustrated are effective antivirals against HEV and their targets within host cells. The figure is adapted from [56] used under a CC BY 4.0 International Licence.

Several compounds have been identified as potential inhibitors of HEV infection. These compounds target various stages of the viral life cycle; however, most of them are confined to basic research and have yet to progress to pre‐clinical studies. Consequently, there is demand for new antiviral agents (Figure 4) [56].

Dr. Klöhn and colleagues discovered that the cathepsin inhibitor K11777 blocks the cleavage of the HEV capsid protein by cathepsins during virus infection, thereby demonstrating antiviral activity against HEV. This inhibitor effectively reduced HEV infection in both hepatoma cells and primary human hepatocytes [57].

In cooperation with KU Leuven, Janssen Pharmaceuticals and Ruhr University Bochum, researchers identified an HEV polymerase inhibitor (JNJ‐9117), which was previously recognised for inhibiting Hepatitis C virus replication. JNJ‐9117 inhibited HEV replication in hepatic and extrahepatic cell lines as well as in primary human hepatocytes. Most strikingly, the inhibitor had a pronounced antiviral effect against HEV in rats, both as prophylactic and therapeutic treatment [58].

Furthermore, Dr. Klöhn presented data on antiviral compound screens consisting of libraries of FDA‐approved compounds and nucleoside/nucleotide analogues to find potential antiviral agents against HEV. This screening led to several promising studies. Notably, the inhibition of the proteins METTL3 or FTO, which are involved in the RNA methylation and demethylation pathways, was found to impede HEV infection. Another compound showing promise as an antiviral agent was the interferon receptor agonist RO8191 which reduces HEV replication and infection in hepatoma cell lines and in primary human hepatocytes.

As HEV is an RNA virus, prone to frequent mutations in its genome, it is likely that effective treatment of HEV infections will require combination therapies.

### 3.4 Risk Factors for Hepatitis E in Kidney Transplant Recipients (By PD Dr. Mira Choi)

Viral infections pose a significant challenge after kidney transplantation. Approximately 17% of the post‐transplant mortality in kidney transplant recipients was caused by infections [59]. Hepatitis is additionally among the top ten causes of mortality after a kidney transplantation [60]. Therefore, it is crucial to prevent an HEV infection in solid organ transplant (SOT) recipients. As SOT recipients receive immunosuppressive therapy to prevent transplant organ rejection, their risk for a chronic HEV infection is increased [61, 9]. In order to characterise HEV infections in kidney transplant recipients, Dr. Choi et al. analysed the course of chronic HEV infections in 17 kidney transplant recipients. They found that most of the HEV infections occur several months or even years after transplantation, when immunosuppressive therapy has been reduced. Additionally, they observed spontaneous remission in only one of the 17 patients and a higher risk for liver fibrosis. There were also signs of extrahepatic manifestations, indicated by the onset of proteinuria [62].

To identify risk factors of an HEV infection in kidney transplant recipients, Dr. Choi and her colleagues continued their research by conducting a study with 312 kidney transplant recipients with elevated liver enzymes, of which 18.9% were positive for HEV. They showed that most (78%) of the HEV‐infected individuals in their study were male and that there was no correlation with other comorbidities. Furthermore, the immunosuppressive regimen had an influence on the risk for chronic HEV infection. It was noticeable that 83% of HEV‐infected individuals were treated with tacrolimus, while only 5.1% received ciclosporin as calcineurin inhibitor treatment. It is important for kidney transplant recipients to avoid raw or undercooked meat or fish to prevent zoonotic infections with HEV. Dr. Choi also highlighted that meat and sausage products also need to be heated to over 70°C for at least 10 min to inactivate HEV. For several products like salami or ham, this might not be the case. Indeed, their study showed that kidney transplant recipients with an HEV infection consumed more pork products like pork steak, salami, or cured pork meat, and there were also more people who touched raw meat with their bare hands in the cohort with an HEV infection compared to the kidney transplant recipients without HEV [63]. These findings are supported by a study where commercially available pork products were tested for HEV RNA, of which 10% tested positive [64].

As a result, Dr. Choi concluded that immunosuppressive therapy after kidney transplantation provides a risk for a chronic HEV infection. In order to avoid such an infection, it is crucial to inform patients about the potential risk of pork products that have not been properly heated to be a source of infection. Hygiene measures during the handling of raw meat, like wearing gloves, should also be considered.

### 3.5 Round Table Discussion: Clinical Management of HEV—Challenge Accepted!

The panellists of the second round table discussed multiple challenges in the clinical management of HEV, which are summarised here in brief.

#### 3.5.1 The Relevance of Antibodies in an HEV Infection

The role of antibodies in the clearance of an HEV infection has yet to be elucidated. Some studies indicated that antibodies could potentially be important in HEV infections. An analysis of Schulz et al. found that patients who are depleted of B‐cells due to a Rituximab treatment may experience a long‐term risk for difficult‐to‐treat chronic HEV. They panelists discussed that this increased risk for chronic hepatitis upon HEV infection might be caused by the Rituximab‐induced hypogammaglobulinemia, but that other conditions that affect the antibody response were not associated with this increased risk. Therefore, further studies are needed to determine the role of antibodies in HEV infections [65]. In another study, SOT recipients were screened for their anti‐HEV IgG at the time of transplantation. After 1 year, HEV infection rates in correlation to the measured anti‐HEV IgG were analysed. In this study, anti‐HEV antibody titres of at least 7 WHO IU/ml seemed to protect individuals from HEV infection. Nevertheless, this does not correlate to antibody titres needed to establish protective immunity [66].

#### 3.5.2 Extrahepatic Manifestations

The mechanisms underlying extrahepatic manifestations of HEV remain unclear. One potential explanation is that the immune response triggered by HEV becomes disseminated, leading to tissue damage beyond the liver [67, 68, 69]. Even though it has been evidenced that HEV can infect human testicular tissue and Sertoli cells and replicate in these cells [70], current reports point to the absence of sexual transmission of HEV [71, 72].

In countries with limited testing capacities for HEV, like Vietnam, the amount of extrahepatic manifestations is underestimated, as there are no studies analysing these complications [73].

#### 3.5.3 Availability of HEV Diagnostics in Other Countries

During the round table discussion, panellists from Nigeria and Vietnam stated that HEV diagnostic tests are not broadly available in their respective countries. This is underscored by the lack of epidemiological data about HEV in Nigeria. The few available studies on the prevalence of HEV in Nigeria indicate high seroprevalence, rendering HEV an underestimated burden in Nigeria. The disease poses a significant public health risk, especially to high‐risk populations, like immunosuppressed individuals, pregnant women, or people living with a chronic HBV infection [74, 75, 76].

A similar situation is present in Vietnam, where HEV is endemic, but the picture of infections is still incomplete. There is no routine testing for HEV available in Vietnam in most hospitals, and the awareness for the disease is largely missing [77]. Additionally, blood donors are not screened for HEV in Vietnam, even though blood donations can be a source of HEV infections, which can be dangerous for risk populations receiving these donations [78].

In order to improve HEV diagnosis in resource‐limited countries, HEV point‐of‐care testing would be beneficial for the detection of HEV infections and hence an improved treatment [79].

#### 3.5.4 Treatment of Patients With an Acute HEV Infection

As most acute HEV infections are self‐limiting, treatment is not required in the majority of cases [7, 6]. In patients with underlying liver disease, HEV infections can cause fulminant hepatitis and lead to severe outcomes [80]. It was therefore debated if an early treatment with Ribavirin is beneficial for patients at risk for severe outcomes. There are currently no clear guidelines on the treatment of patients with acute HEV infection, who are at risk of developing fulminant hepatitis. A retrospective, single‐center, observational study in Germany did not observe improved HEV clearance rates in non‐cirrhotic patients under immunosuppression in patients who received an early ribavirin treatment. Nevertheless, early administration of ribavirin might have shortened the duration of viremia [81]. Meanwhile, some case reports have demonstrated the efficacy and safety of ribavirin in treating patients with chronic liver disease, who presented with an acute HEV infection [82]. Due to the limited evidence on the advantages of an early ribavirin treatment for patients with an underlying immunosuppression or chronic liver disease, the EASL and the British Transplantation Society mention in their guidelines on HEV infections that an early treatment of acute HEV infections with ribavirin in at‐risk patients might be beneficial [7, 80].

## 4 Discussion

The symposium compellingly highlighted that hepatitis E virus (HEV) infections have emerged as a major global health concern. In Africa and Southeast Asia, transmission primarily occurs via the faecal‐oral route, emphasising the ongoing need for improved hygiene and sanitation infrastructure. In contrast, in Europe and North America, HEV infections are mainly foodborne, with undercooked meat recognised as the principal source. In addition, zoonotic rat HEV is widely distributed in wild rat populations and can be sporadically transmitted to humans, adding further complexity to the epidemiological landscape.

Substantial progress has been made in recent years in unravelling HEV biology, including mechanisms of replication, host interaction, and immune evasion. Nevertheless, ribavirin remains the only therapeutic option available to date although its use is limited to off‐label applications. It was therefore encouraging to see multiple groups presenting novel antiviral strategies targeting various stages of the HEV life cycle. These developments hold great promise for the improvement of diagnostic tools for early‐stage infection, as well as for the establishment of targeted and effective treatment regimens.

Throughout the meeting, critical challenges in the clinical management of HEV became apparent. These include the urgent need for specific antiviral agents, effective prophylactic measures and the expansion of diagnostic infrastructure, particularly in resource‐limited settings.

Importantly, the symposium underscored the vital role of interdisciplinary and international collaboration in addressing these challenges. By bringing together researchers from across the globe, this meeting fostered fruitful scientific exchange and initiated new collaborations. It laid a strong foundation for continued progress in the field of HEV research. Building on this momentum, the next international HEV meeting is planned for 2027. We look forward to continuing this important dialogue and driving innovation through collective effort and shared expertise.

## Author Contributions

J.K., H.W. and D.T. planned, organised and hosted the conference. L.B. and D.T. wrote the original draft. All authors contributed to their respective chapters and prove read and approved the final draft.

## Conflicts of Interest

The authors declare no conflicts of interest.

## Data Availability

For this conference report, no new data was produced. All relevant data is published together with the referenced articles.
